# Exacerbation of atrial fibrillation related to dulaglutide use

**DOI:** 10.1002/ccr3.4223

**Published:** 2021-05-15

**Authors:** Jayden Lee, Idopise E. Umana, Judy Nguyen

**Affiliations:** ^1^ School of Pharmacy, Philadelphia College of Osteopathic Medicine – Georgia Suwanee GA USA; ^2^ Graduate Medical Education Internal Medicine Residency Program Northeast Georgia Medical Center Gainesville GA USA; ^3^ Philadelphia College of Osteopathic Medicine – Georgia Suwanee GA USA

**Keywords:** atrial fibrillation, dulaglutide, GLP‐1 receptor agonist

## Abstract

Dulaglutide is associated with sinus tachycardia, increased PR interval, and 1st degree AV block. These conduction abnormalities can increase the risk of arrhythmia. Dulaglutide should be used with caution in patients with pre‐existing arrhythmia.

## Graphical abstract

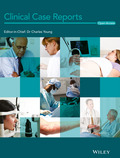

## 1 INTRODUCTION

Dulaglutide is a commonly used, injectable drug for type 2 diabetes associated with sinus tachycardia, increased PR interval, and 1st degree AV block. These adverse effects related to cardiac conduction can increase the risk of arrhythmia. Thus, dulaglutide should be used with caution in patients with pre‐existing arrhythmia.Atrial fibrillation (AF) is the most common cardiac arrhythmia defined as having irregularly irregular heart rhythm and rate. It is triggered by various electrophysiological changes in atria, resulting in complex conduction patterns and rapid atrial rate. The common risk factors associated with AF include hypertension, cardiovascular disease, heart failure, diabetes, and increasing age.^1^

Dulaglutide is a human glucagon‐like peptide‐1 receptor (GLP‐1) agonist indicated for patients with type 2 diabetes mellitus (T2DM), given as a once‐weekly subcutaneous injection. The common adverse reactions of dulaglutide include gastrointestinal effects, such as nausea, vomiting, and diarrhea. These adverse reactions are dose‐dependent, with prevalence peaking in the first two weeks and stabilizing in 6 to 8 weeks. Other adverse events include pancreatitis, sinus tachycardia, and hypoglycemia (typically combined with insulin or sulfonylureas).^2^

The Assessment of Weekly Administration of LY2189265 (dulaglutide) in Diabetes (AWARD) trials were phase 2 and 3 trials, comparing dulaglutide's safety and efficacy with exenatide, insulin glargine, metformin, sitagliptin, and liraglutide. Overall, these trials demonstrated that dulaglutide reduced blood pressure (BP), increased heart rate (HR), and increased PR interval.^3^, ^4^, ^5^, ^6^, ^7^, ^8^

Furthermore, a randomized controlled trial compared dulaglutide (0.75 or 1.5 mg) with placebo and evaluated ambulatory BP and HR for 26 weeks. The dulaglutide 1.5 mg group demonstrated a reduction in BP and an increase in HR. Three adverse events of sinus tachycardia were reported in the dulaglutide groups as well.^9^

Based on the literature review, the use of dulaglutide has been associated with increased HR, decreased BP, and increased PR interval. Although dulaglutide appears to have questionable effects on cardiac conduction, no literature to our knowledge has found an association between dulaglutide and AF exacerbation. The purpose of this case report is to present a patient case of AF exacerbation after a single administration of dulaglutide 0.75 mg in a woman with a history of T2DM and pre‐existing paroxysmal AF.

## 2 CASE PRESENTATION

A 75‐year‐old Caucasian woman with a history of T2DM, hypertension, anxiety, obesity, chronic obstructive pulmonary disease, coronary artery disease, congestive heart failure, and obstructive sleep apnea developed paroxysmal AF and received her first cardioversion in 2017. Initially, she was managed with flecainide, rivaroxaban, and metoprolol, but her cardiologist soon discontinued metoprolol due to symptomatic bradycardia with HR in the 40s and fatigue. After stopping the metoprolol, the follow‐up electrocardiogram (ECG) revealed normal sinus rhythm with HR at 65 bpm. At this time, the thyroid‐stimulating hormone level was also within the normal range.

Two months later, she presented with complaints of increased appetite, weight gain, symptomatic hypoglycemia, and fluctuating fasting blood glucose (FBG) levels, ranging from the 70s up to 300s mg/dL. At that time, the treatment with metformin, glimepiride, and insulin degludec resulted in an A1c of 8.7%. Her primary care team discontinued glimepiride as a probable cause of her hypoglycemic episodes, and dulaglutide 0.75 mg once weekly was initiated. Since buspirone was suspected of causing increased appetite, the anxiety treatment was changed from buspirone to duloxetine 60 mg once daily.

Six days later, after a single subcutaneous administration of dulaglutide 0.75 mg, she arrived at the emergency department (ED) with complaints of severe nausea, intermittent vomiting, weakness, poor appetite, and palpitations. She reported that she started vomiting the night of dulaglutide injection and, since then, had nausea, weakness, and poor appetite. She felt palpitations and vague atypical chest discomfort over the past few days prior to presentation, making her visit the ED. She has been checking her FBG levels during this time, and they have ranged from 100 to 120 mg/dL with no hypoglycemia episode.

On evaluation, she was volume‐depleted and dehydrated, which resulted in acute kidney injury. Thus, the hospitalist held furosemide, lisinopril, and potassium supplement. Chemistry showed elevated creatinine, glucose, and blood urea nitrogen (BUN) levels but normal sodium, potassium, calcium, and magnesium levels. Troponin test was unremarkable as well. Initial ECG showed sinus tachycardia, but subsequent ECG showed rapid AF with HRs in the 120s. She was initially managed with intravenous (IV) fluid, labetalol, and diltiazem, which improved BP and HR in the ED. She was then monitored on telemetry, and serum creatinine improved to be almost at baseline. HR remained stable with IV fluids and home flecainide; thus, oral beta‐blocker was not continued. Nausea and vomiting completely resolved, and she was discharged without a beta‐blocker after a 2‐day stay in a stable condition with euvolemic status. As the cause of severe nausea and vomiting, dulaglutide was discontinued upon discharge.

Two days later, she presented to the ED again with palpitation, weakness, and shortness of breath. At this time, nausea and vomiting were not present. A comprehensive metabolic panel this time showed all normal levels, including glucose, creatinine, and BUN. Troponin test was negative again, and magnesium was within the normal range. An echocardiogram was performed, showing a left ventricular ejection fraction of 55%‐60% with mild concentric hypertrophy on the left ventricle. ECG again showed AF with a rapid ventricular response with an HR of 126 bpm. She was managed with a diltiazem drip since her HR kept rebounding high after two doses of IV diltiazem. She was then admitted to the cardiology floor, monitored on telemetry, and underwent cardioversion (2 shocks), resulting in normal sinus rhythm with HR of 71 bpm. The cardiologist doubled the dose of flecainide and restarted low‐dose metoprolol (12.5 mg twice daily). She was discharged the next day in a stable condition.

## 3 OUTCOME AND FOLLOW‐UP

At postdischarge follow‐up, she still reported some nausea; thus, duloxetine was reduced to 30 mg daily, which resolved nausea. BG levels were also controlled at this time without a GLP‐1 agonist. During her electrophysiology consultation, she reported experiencing fatigue with ECG showing bradycardia at 41 bpm, and metoprolol was discontinued again. On the follow‐up ECG one week later, normal sinus rhythm was maintained at HR of 71 bpm with symptomatic improvement. The summary of events is presented in Table 1.

**TABLE 1:** Pertinent heart rate, blood pressure, and electrocardiogram findings

**Date**	**Time**	**BP (mmHg)**	**HR (bpm)**	**ECG**	**PR Interval (ms)**	**Comment**
**8 May 2018**	‐	110/68	45	Sinus bradycardia with nonspecific T abnormalities	189	Metoprolol discontinued
**16 May 2018**	‐	142/82	75	Normal sinus rhythm	202	‐
**17 July 2018**	‐	124/70	66	‐	‐	Regular rhythm per PE
**20 July 2018**	‐	N/A	N/A	‐	‐	Dulaglutide 0.75 mg SC administered
**26 July 2018**	08:11	149/116	124	‐	‐	Presented at ED
	08:14	‐	129	Sinus tachycardia with nonspecific T abnormalities	128	‐
	09:00	111/63	117	‐	‐	‐
	09:18	‐	117	AF with nonspecific T abnormalities	‐	IV labetalol and diltiazem given
	09:30	121/85	111	‐	‐	‐
	10:00	111/75	109	‐	‐	‐
	10:30	115/79	69	‐	‐	‐
	11:00	117/81	80	‐	‐	‐
	12:00	160/110	88	‐	‐	Sent to be monitored on telemetry
**27 July 2018**	10:46	128/72	74	‐	‐	‐
	15:19	132/73	65	‐	‐	‐
**28 July 2018**	03:00	142/79	95	‐	‐	‐
	08:36	119/87	106	‐	‐	Discharged
**31 July 2018**	07:27	145/98	130	‐	‐	Presented at ED
	07:29	‐	126	AF with RVR	‐	Metoprolol initiated
	08:15	126/84	96	‐	‐	‐
	09:15	110/74	107	‐	‐	‐
	10:30	106/68	102	‐	‐	‐
	13:28	‐	85	AF	‐	‐
	15:06	‐	59	Sinus bradycardia	204	Status post‐DCCV
**1 August 2018**	04:00	154/74	71	‐	‐	‐
	05:56	‐	69	Normal sinus rhythm	191	‐
	07:12	162/73	72	‐	‐	Discharged
**6 August 2018**	‐	‐	‐	‐	‐	Duloxetine dose reduced
**8 August 2018**	‐	130/62	53	‐	‐	At hospital follow‐up with PCP
**15 August 2018**	‐	130/64	41	Sinus bradycardia	‐	Metoprolol discontinued
**23 August 2018**	‐	136/72	71	Normal sinus rhythm	210	‐

Abbreviations: BP, blood pressure; HR, heart rate; ECG, electrocardiogram; PE, physical examination; ED, emergency department; AF, atrial fibrillation; SC, subcutaneously; RVR, rapid ventricular response; DCCV, direct current cardioversion; PCP, primary care provider.

## 4 DISCUSSION

The cause of AF exacerbation was suspected to be due to volume depletion secondary to dulaglutide's vomiting side effect. However, AF persisted even after vomiting was resolved and volume status was corrected, requiring the second hospitalization and cardioversion. The AWARD trials and the study evaluating the effect of dulaglutide on ambulatory BP and HR demonstrated that dulaglutide was typically associated with an increase in HR by 3‐4 bpm.^3^, ^4^, ^5^, ^6^, ^7^, ^8^, ^9^ On the contrary, this patient's HR increased by approximately 60 bpm, which was significantly higher than the expected increase seen in the clinical trials.

The half‐life of dulaglutide is approximately five days.^2^ A drug typically requires about four half‐lives for the drug's amount in the blood circulation to be negligible, which means dulaglutide's effect may last up to 20 days. In this case, all the cardiac‐related events occurred during these 20 days since the dulaglutide administration.

Another GLP‐1 agonist, albiglutide, has already been associated with AF. A meta‐analysis of phase 2b and three trials evaluated the cardiovascular safety of albiglutide. Although the meta‐analysis found no significant difference between albiglutide and comparators (glimepiride, insulin glargine, insulin lispro, liraglutide, pioglitazone, or sitagliptin) in cardiovascular death, nonfatal myocardial infarction, or nonfatal stroke, the albiglutide groups were associated with AF or atrial flutter.^10^ Similarly, a systematic review of GLP‐1 agonists found a correlation between the incidences of AF with albiglutide. Other GLP‐1 agonists, including dulaglutide, were not viewed as having an increased risk of AF. However, the authors noted that the studies reviewed in this meta‐analysis did not include AF as a safety outcome.^11^

GLP‐1 receptors are found in the human pancreas and gut as well as in the human heart. They are expressed in all four cardiac chambers but localized at the sinoatrial node.^12^ An animal study demonstrated that the activation of GLP‐1 receptors in the autonomic nervous system enhances sympathetic nervous system activity and reduces parasympathetic nervous system activity. Also, atrial GLP‐1 receptors contribute to the control of HR.^13^ One may theorize that over‐activation of cardiac GLP‐1 receptors may increase the risk of AF exacerbation in patients with pre‐existing paroxysmal AF, who may be sensitive to GLP‐1 activation.

Furthermore, a study involving over 288,000 participants evaluated the association between prolonged PR interval and AF. The study concluded that longer PR intervals were associated with an increased risk of AF.^14^ Because dulaglutide is associated with PR interval prolongation, it is possible to suspect that dulaglutide may increase the risk of AF by prolonging the PR interval. On this note, the Canadian drug monograph of dulaglutide warns about the association between PR interval prolongation and increased risk of incident AF and recommends caution in patients with AF history.^15^ However, this case showed the opposite, with a significant drop in PR interval after dulaglutide administration. The case again highlights the fact that the effect of dulaglutide on cardiac conduction is still unclear.

On the other hand, it is possible that the natural history of paroxysmal AF took its course and resulted in a coincidental recurrence of AF. The patient's age and hypertension are risk factors for developing recurrent AF. Several studies have evaluated the recurrence of paroxysmal AF but with varying degrees of recurrence rates. One study reported a 50% recurrence rate of paroxysmal AF at 12‐month follow‐up after successful cardioversion.^16^ Thus, it is difficult to rule out the coincidental, natural AF recurrence.

Another limitation of this case report is the simultaneous initiation of dulaglutide and duloxetine. Duloxetine increases serotonin and norepinephrine effects, which may cause nausea and palpitation, respectively.^17^ Nonetheless, duloxetine is not associated with AF. Besides, the patient was continued on duloxetine and was not experiencing sinus tachycardia or other types of arrhythmias.

The Researching Cardiovascular Events with a Weekly Incretin in Diabetes (REWIND) trial evaluated dulaglutide's cardiovascular safety compared to placebo in patients with T2DM. The trial results showed dulaglutide reduced the risk of the primary composite outcome, which consisted of nonfatal myocardial infarction, nonfatal stroke, and death from cardiovascular causes or unknown causes.^18^ The trial also measured prespecified adverse events of particular interest, including supraventricular tachycardia or cardiovascular conduction disorders. The study reported that supraventricular tachycardia or cardiovascular conduction disorders occurred more in the dulaglutide group than the placebo group, but it was not statistically significant (4.4% vs. 3.9%, *P* =.26).^18^ Unfortunately, the authors provided no breakdown of different types of arrhythmias seen in the trial. Also, there was no subgroup analysis completed on patients with pre‐existing paroxysmal AF.

Currently, available clinical trials lack evidence on dulaglutide and its potential risk of AF. Based on the scientific data discussed here, it appears that dulaglutide certainly affects cardiac conduction but with uncertain consequences. Further investigation is needed to evaluate the risk of arrhythmia with dulaglutide use.

## CONFLICT OF INTEREST

All authors have no conflict of interest to disclose.

## AUTHOR CONTRIBUTIONS

Jayden Lee, PharmD, BCACP, CACP (corresponding author), Jayden Lee: involved in the primary and corresponding author of this manuscript, the clinical pharmacist who managed this patient's diabetes, initiated dulaglutide, investigated this case, also responsible for the literature review, and wrote a significant portion of the manuscript. Idopise E. Umana, MD; Idopise Umana: involved in the patient's primary care physician, managed the patient for all her medical conditions for years, and also reviewed the manuscript before submission. Judy Nguyen, PharmD; Judy Nguyen,a student pharmacist at the time of this case, but now graduated: assisted the primary author with managing the patient and putting the relevant case information together from a chart review.

## ETHICAL APPROVAL

Hereby, I, Jayden Lee, consciously assure that for the manuscript “Exacerbation of atrial fibrillation related to dulaglutide use” the following is fulfilled: 1) This material is the authors' own original work, which has not been previously published elsewhere. 2) The paper is not currently being considered for publication elsewhere. 3) The paper reflects the authors' own research and analysis in a truthful and complete manner. 4) The paper properly credits the meaningful contributions of coauthors. 5) All sources used are properly disclosed (correct citation). 6) All authors have been personally and actively involved in substantial work leading to the paper and will take public responsibility for its content.

## Data Availability

The data that support the findings of this study are available on request from the corresponding author. The data are not publicly available due to privacy or ethical restrictions.
